# Sequential and synergistic delivery of lipiodol and drug-eluting microspheres circumvents incompatibility to enhance targeted chemoembolization

**DOI:** 10.3389/fphar.2026.1800481

**Published:** 2026-03-30

**Authors:** Nan Du, Wen Zhang, Chao Chen, Ying Wang, Xinhong He, Jianjun Luo, Zhiping Yan, Wen-Tao Li

**Affiliations:** 1 Department of Interventional Radiology, Fudan University Shanghai Cancer Center, Shanghai, China; 2 Department of Oncology, Shanghai Medical College, Fudan University, Shanghai, China; 3 Zhongshan Hospital Fudan University, Shanghai, China

**Keywords:** chemoembolization, epirubicin, hepatocellular carcinoma, lipiodol, liver function, microsphere, rabbit, tumor necrosis

## Abstract

**Background:**

Conventional (cTACE) and drug-eluting bead transarterial chemoembolization (D-TACE) are limited by physicochemical incompatibility when lipiodol and microspheres are mixed. This study evaluated a sequential co-delivery strategy (M-TACE) designed to synergize these agents in a rabbit VX2 liver tumor model.

**Methods:**

Animals were randomized into control, cTACE, D-TACE, and M-TACE (epirubicin-lipiodol emulsion followed by epirubicin-loaded microspheres) groups. Primary outcomes included pharmacokinetics, tumor necrosis, and lipiodol distribution.

**Results:**

The cTACE group showed a higher systemic peak epirubicin concentration (C ∼ max∼: 276.08 ± 41.74 ng/mL) than D-TACE (79.37 ± 16.26 ng/mL) and M-TACE (92.13 ± 12.68 ng/mL) (p < 0.001), while total 24-h exposure (AUC0–24 h) was comparable. M-TACE achieved intratumoral drug concentrations equivalent to D-TACE at day 1 and 14, confirming unimpaired microsphere elution. M-TACE induced superior tumor necrosis (95.4% ± 6.7%) versus cTACE (85.1% ± 6.2%) and D-TACE (88.6% ± 8.4%) (p < 0.001), with less peritumoral liver necrosis than D-TACE (50.9% ± 6.0% vs. 61.7% ± 9.0%, p = 0.039). M-TACE also yielded more focal lipiodol deposition (72.2% ± 7.4% tumor coverage) with reduced parenchymal extravasation versus cTACE (92.9% ± 7.8%; p < 0.001).

**Conclusion:**

The sequential M-TACE strategy successfully decouples lipiodol and microsphere delivery, circumvents the practical consequences of incompatibility while harnessing synergistic advantages—lipiodol’s capillary blockade and microspheres’ sustained drug release—resulting in enhanced efficacy and favorable local safety profile.

## Highlights

- Technical Innovation: Overcoming Core Material IncompatibilityThis study develops a sequential co-delivery strategy (M-TACE) that circumvents the practical consequences of incompatibility between lipiodol emulsion and drug-eluting microspheres by temporal separation rather than physical mixing. The protocol delivers lipiodol first for capillary-level blockade, followed by microspheres for sustained chemotherapy, achieving synergistic efficacy. Pharmacokinetics confirm unimpaired drug elution from microspheres, providing a feasible technical pathway for combination therapy.
- Mechanistic Edge: Unique “Low Systemic, High Local” Pharmacokinetic ProfileM-TACE exhibits a distinct pharmacokinetic advantage: it achieves systemic drug exposure (AUC) comparable to cTACE but with a significantly lower peak plasma concentration (Cmax), indicating reduced potential systemic toxicity. Crucially, it maintains intratumoral drug levels equivalent to D-TACE, establishing an ideal “low systemic, high local” drug distribution profile that underlies its potent antitumor effect.
- Efficacy & Safety: Superior Tumor Control with an Improved Therapeutic WindowIn the rabbit VX2 model, M-TACE induced the highest tumor necrosis rate (95.4%), achieved complete metabolic remission on PET/CT, and reduced metastatic burden. Its efficacy was independent of uniform initial lipiodol deposition, overcoming the limitation of marginal recurrence in conventional TACE. Furthermore, the strategy used less lipiodol and caused less peritumoral liver injury, demonstrating a favorable therapeutic window for clinical translation.

## Introduction

Transarterial chemoembolization (TACE) remains a cornerstone in the management of intermediate-stage hepatocellular carcinoma (HCC) (Reig et al., 2025). The two principal technical approaches—conventional TACE (cTACE) with lipiodol-based emulsions and drug-eluting bead TACE (D-TACE)—rely on distinct yet complementary pharmacologic principles. cTACE utilizes lipiodol to achieve capillary-level blockade and drug deposition deep within the tumor parenchyma and portal venules, providing a critical imaging surrogate for treatment margins (Casadaban et al., 2016). Conversely, D-TACE employs calibrated microspheres to provide sustained, localized release of chemotherapeutic agents, minimizing systemic exposure (Guo et al., 2025). Despite their individual merits, clinical practice and meta-analyses have not established a definitive survival advantage of one technique over the other, highlighting an unmet need for therapeutic optimization (Fuchs et al., 2017; Ikeda et al., 2022; Zhou et al., 2024).

A fundamental, yet under-addressed, limitation lies in the physicochemical and mechanical incompatibility of lipiodol and microspheres when used concurrently. Lipiodol, as an oily emulsion, can coat and potentially clog the porous structure of drug-eluting beads, impairing their drug-elution kinetics and homogeneous distribution (Casadaban et al., 2016). Conversely, the simultaneous administration of microspheres may disrupt the formation and distal penetration of a stable lipiodol emulsion (Hong et al., 2006; Yamamoto et al., 2006). This incompatibility forces a clinical trade-off: cTACE offers superior distal penetration but suffers from rapid lipiodol “washout” and inconsistent drug dosing, while D-TACE provides controlled release but may fail to reach the tumor’s most distal microvasculature, potentially leaving therapeutic blind spots (Chuang et al., 2023; Clouet et al., 2014).

We hypothesize that a temporally sequenced, rather than concurrent, delivery of these agents could circumvent their incompatibility while synergizing their mechanistic strengths. This study introduces and evaluates a sequential co-delivery strategy, termed M-TACE, in a preclinical VX2 rabbit liver tumor model. The approach first administers a lipiodol-epirubicin emulsion to saturate the tumor microcirculation and portal drainage routes, establishing an initial embolic and chemotherapeutic effect. Following a brief interval to allow for initial deposition, drug-eluting microspheres are then infused to occlude the proximal tumor-feeding arterioles, thereby securing the lipiodol depot and instituting a second phase of sustained drug release. The sequential M-TACE protocol differs from prior attempts to combine lipiodol and beads. It is distinct from co-administration in a single mixture (limited by incompatibility) and from separate treatment sessions. The novelty lies in the precise, single-session temporal order: lipiodol first for distal saturation, followed shortly by microspheres.

This study was therefore designed to test the central premise that temporally segregating the administration of lipiodol and drug-eluting microspheres can overcome their incompatibility and harness their synergistic potential to improve TACE outcomes.

## Materials and methods

### Experimental animal model

All animal experiments received approval from our hospital’s Institutional Animal Care and Use Committee and were conducted in strict accordance with ARRIVE guidelines. New Zealand White rabbits (3.0–3.5 kg) served as the experimental subjects. The VX2 carcinoma strain was maintained by serial transplantation in the hind limb of donor animals. For model establishment, freshly harvested VX2 tumor tissue was minced into ≈1 mm^3^ fragments and surgically implanted into the left hepatic lobe of recipient rabbits under general anesthesia (induced via intramuscular injection of ketamine [30 mg/kg] and xylazine [5 mg/kg]). Tumors were allowed to grow for 2–3 weeks. Ultrasound was used to monitor growth, and lesions measuring approximately 1.0 cm in diameter were deemed suitable for embolization therapy.

### Study Design and Randomization

Tumor-bearing rabbits were randomly allocated into four experimental groups (n = 8 per group): (1) Control (intra-arterial normal saline); (2) cTACE (conventional TACE using an epirubicin-lipiodol emulsion); (3) D-TACE (drug-eluting bead TACE using epirubicin-loaded HepaSphere™ microspheres); and (4) M-TACE (sequential TACE: epirubicin-lipiodol emulsion followed by epirubicin-loaded microspheres). Randomization was performed using a computer-generated sequence.

### Preparation of embolic agents

The epirubicin-lipiodol emulsion was prepared by mixing epirubicin solution (12 mg/mL) with lipiodol at a 1:2 (v:v) ratio via a three-way stopcock. HepaSphere™ microspheres (20–40 μm dry state; Merit Medical) were loaded with epirubicin according to the manufacturer’s instructions. Briefly, 50 mg of epirubicin was dissolved in 20 mL of normal saline and mixed with the microspheres to achieve a target loading of 4 mg per treatment dose. Gelatin sponge particles (150–350 μm) were prepared as needed for supplemental embolization.

### Transarterial chemoembolization procedure

All procedures were performed under anesthesia. Selective catheterization of the tumor-feeding left hepatic artery was achieved using a 1.9-Fr microcatheter. The cTACE group received 1 mL epirubicin-lipiodol emulsion (4 mg epirubicin); the D-TACE group received microspheres loaded with 4 mg epirubicin according to the manufacturer’s protocol and technical recommendations; the M-TACE group received 0.2 mL of the emulsion followed by microsphere injection after a 5-min interval, with a total epirubicin dose of 4 mg. Gelatin sponge particles were used for supplemental embolization if needed in three TACE groups. Gelatin sponge particles (150–350 μm) were used only when angiographic near-stasis could not be achieved with the primary embolic agents alone. The criteria for gelatin sponge use were: persistence of antegrade flow in the target artery after complete administration of the primary embolic dose, or visualization of residual tumor blush on control angiography. Embolization endpoints were standardized to near-stasis. Control animals underwent angiography and injection of an equivalent volume of normal saline.

### Pharmacokinetic and tissue drug concentration analysis

A subgroup of animals (n = 3 per TACE group) was euthanized 24 h post-procedure for analysis of early intratumoral drug concentration. The remaining animals (n = 5 per TACE group) were maintained for the 14-day terminal endpoint.

Systemic pharmacokinetics were evaluated by collecting arterial blood samples at baseline, 10, 30, 60, 180 min, and 24 h post-embolization. Plasma was separated and stored at −80 °C. Epirubicin concentrations in plasma and in homogenized tumor tissue (harvested at 1 and 14 days) were quantified using a validated high-performance liquid chromatography-tandem mass spectrometry (HPLC-MS/MS) method, as previously described (Ji et al., 2024). The lower limit of quantification was 5 ng/mL. Non-compartmental analysis was performed using GraphPad Prism (v8.0). The maximum plasma concentration (Cmax) and time to Cmax (Tmax) were recorded directly from observed data. The area under the plasma concentration-time curve from 0 to 24 h (AUC_0–24h_) was calculated using the linear trapezoidal rule, assuming a baseline concentration of zero. Intratumoral epirubicin concentration was expressed as ng per gram of wet tumor tissue (ng/g).

### Longitudinal assessments: Liver function, tumor volume, and metabolic activity

Blood samples for liver function tests (alanine aminotransferase [ALT], aspartate aminotransferase [AST], total bilirubin [TB]) were collected at baseline, 1, 7, and 14 days post-procedure.

Tumor volumes were quantified at baseline and at the 14-day endpoint prior to sacrifice. Volumetric analysis was performed using the high-resolution CT component of the PET/CT scans. Volumes were calculated by manual segmentation of the tumor contour on all axial slices. The percentage change in volume from baseline to day 14 was calculated for each animal.

Metabolic activity was evaluated using ^18^F-fluorodeoxyglucose positron emission tomography/computed tomography (^18^F-FDG PET/CT) at baseline and day 14. Images were co-registered, and a volume of interest was drawn around the entire tumor on the CT scan to derive the maximum standardized uptake value (SUVmax). Complete metabolic suppression was defined as the absence of visually detectable focal ^18^F-FDG activity, with a corresponding tumor SUVmax that did not exceed 2.5 (approximating the upper limit of normal liver background).

### Histopathological and immunohistochemical quantification

At the terminal endpoint (day 14), liver and tumor tissues were harvested, fixed, and processed for histological analysis. Hematoxylin and eosin (H&E)-stained sections were digitally scanned, and the percentage of tumor necrosis was calculated as (necrotic area/total tumor area) × 100% by two blinded pathologists. Lipiodol distribution was visualized and quantified on Oil Red O-stained frozen sections.

Immunohistochemistry was performed for hypoxia-inducible factor 1-alpha (HIF-1α), vascular endothelial growth factor (VEGF), cluster of differentiation 31 (CD31), and proliferating cell nuclear antigen (PCNA). Stained sections were digitized, and a semi-quantitative integral optical density (IOD) for each marker was calculated across a minimum of three representative high-power fields (×200 magnification) per tumor using Image-Pro Plus 6.0 software (Media Cybernetics, Bethesda, MD, United States), with consistent color thresholding applied. The animal experiment flowchart is shown in Supplementary Figure S3.

### Statistical analysis

Data are presented as mean ± standard deviation or median with interquartile range, as appropriate. Differences between groups were performed using one-way ANOVA followed by Tukey’s *post hoc* test, or the Kruskal–Wallis test with Dunn’s *post hoc* test for non-parametric data. Intra-group comparisons (e.g., tumor volume change) were assessed using paired t-tests or Kruskal–Wallis test. *p* < 0.05 was considered as significant. All analyses were conducted using SPSS version 22.0, and a two-sided p-value <0.05 was considered statistically significant.

## Results

### Technical success and procedural outcomes

**FIGURE 1:**
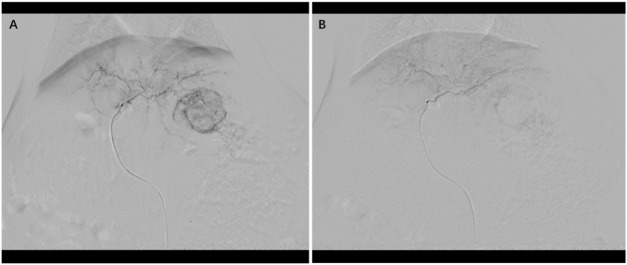
Representative procedural angiography. Digital subtraction angiography images illustrate the technical success of embolization. **(A)** Pre-embolization angiogram showing hypertrophic tumor vasculature. **(B)** Post-embolization angiogram demonstrating successful occlusion of the tumor-feeding artery and cessation of tumor blush, confirming the endpoint of near-stasis.

The drug loading efficiency of epirubicin onto HepaSphere™ microspheres was determined by measuring the residual epirubicin concentration in the loading supernatant using high-performance liquid chromatography, was calculated to be 99%. As shown in Supplementary Figure S1, the loaded microspheres exhibited a uniform spherical morphology with an average diameter of 84.2 ± 17.6 μm, a size range selected to target the terminal tumor vasculature. The morphological characteristics and size distribution of the epirubicin-loaded microspheres were analyzed using light microscopy. The microspheres maintained a uniform spherical shape post-loading. All transarterial chemoembolization (TACE) procedures were successfully completed according to the predefined protocol, achieving angiographic endpoint of near-stasis in the target left hepatic artery. Gelatin sponge was used in 2/8 animals in cTACE group, 1/8 in D-TACE group, and 1/8 in M-TACE group. Representative digital subtraction angiography images are provided in Figure 1. To facilitate early pharmacokinetic analysis, three animals from each TACE group were euthanized 24 h post-procedure. Therefore, the subsequent longitudinal assessments included five animals per TACE group and eight animals in the control group.

### Pharmacokinetics and intratumoral drug delivery

**FIGURE 2:**
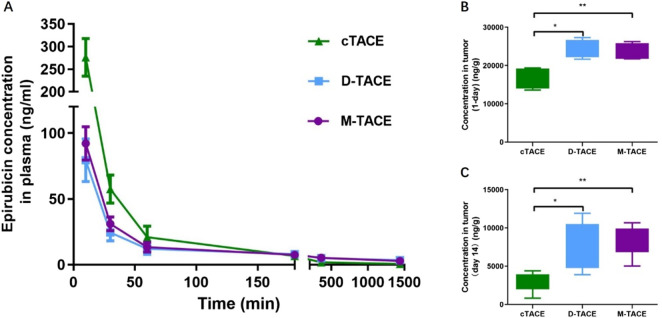
Pharmacokinetic profile and intratumoral epirubicin concentration. **(A)** Mean plasma concentration-time curves of epirubicin within the 24 h post-TACE. The area under the plasma concentration-time curve (AUC∼0–24 h∼), was comparable among all three TACE protocols (cTACE: 9201.88 ± 1276.80 ng/mL•min; D-TACE: 9388.71 ± 1581.08 ng/mL•min; M-TACE: 9523.85 ± 1021.0 ng/mL•min; *p* = 0.581). The cTACE group shows a significantly higher initial peak. **(B,C)** Box plots of intratumoral epirubicin concentration at day 1 **(B)** and day 14 **(C)**. Data are presented as mean ± SD; intergroup differences were analyzed by one-way ANOVA followed by Tukey’s *post hoc* test (*p < 0.05). PK data: n = 3/group at 24 h; intratumoral: n = 3/group (Day1), n = 5/group (Day14).

Analysis of systemic epirubicin exposure revealed distinct pharmacokinetic profiles (Figure 2). The time to reach Cmax (Tmax) was 10 min for all TACE groups. The cTACE group exhibited a sharp, high plasma peak concentration (Cmax: 276.08 ± 41.74 ng/mL), which was significantly greater than the sustained, lower profiles observed in D-TACE (79.37 ± 16.26 ng/mL) and M-TACE (92.13 ± 12.68 ng/mL) groups (p < 0.001). The total systemic exposure over 24 h, measured as the area under the plasma concentration-time curve (AUC∼0–24 h∼), was comparable among all three TACE protocols (cTACE: 9201.88 ± 1276.80 ng/mL•min; D-TACE: 9388.71 ± 1581.08 ng/mL•min; M-TACE: 9523.85 ± 1021.0 ng/mL•min; *p* = 0.581). In contrast to the systemic exposure, intratumoral drug concentrations demonstrated a fundamentally different pattern (Table 1). At day 1, intratumoral epirubicin concentrations were comparable between the M-TACE (23,627.96 ± 2,210.76 ng/g) and D-TACE (24,370.31 ± 2,338.43 ng/g) groups (p = 0.678), both of which were significantly higher than those in the cTACE group (16,726.72 ± 2,766.47 ng/g, p < 0.01). This significant advantage in localized drug retention persisted at day 14, with concentrations in the M-TACE and D-TACE groups remaining approximately threefold higher than in the cTACE group (p < 0.01).

**TABLE 1:** Plasma and intratumoral pharmacokinetic parameters of epirubicin.

​	cTACE group	D-TACE group	M-TACE group	*p* value
Cmax (ng/mL)	276.08 ± 41.74	79.37 ± 16.26	92.13 ± 12.68	<0.001*
AUC_0-24h_ (ng*min/mL)	9201.88 ± 1276.80	9388.71 ± 1581.08	9523.85 ± 1021.01	0.581
Intratumoral concentrations (mean ± SD) at 1 day (ng/g)	16726.72 ± 2766.47	24370.3 ± 2338.43	23627.96 ± 2210.76	0.002*
Intratumoral concentrations at 14-day(ng/g)	2740.13 ± 1241.96	7758.08 ± 3118.06	7495.40 ± 2876.93	0.001*

Cmax: maximum plasma concentration; AUC_0-24h_: area under the plasma concentration-time curve from 0 to 24 h *p < 0.05 indicates a significant difference.

### Systemic safety and tumor growth dynamics

As expected from the embolic insult, plasma alanine aminotransferase (ALT) and aspartate aminotransferase (AST) levels peaked at day 1 across all TACE groups before trending toward baseline by day 14 (Table 2). The initial hepatocyte injury was most pronounced in the D-TACE group, which showed significantly higher ALT levels at day 1 compared to both the cTACE and M-TACE groups (p < 0.001) (Supplementary Figure S2). Total bilirubin levels showed a mild, transient elevation post-procedure.

**TABLE 2:** Temporal changes in liver function indices following intervention.

​	Control	cTACE	D-TACE	M-TACE	F value	*p* value
ALT (U/L)						
Pre-	36.1 ± 4.1	35.2 ± 4.2	35.1 ± 3.4	35.2 ± 3.4	0.115	0.950
Day 1	96.1 ± 30.8	519.5 ± 110.1	837.1 ± 179.9	619.9 ± 139.4	47.713	<0.001^*^
Day 7	41.9 ± 4.9	119.1 ± 37.0	158.1 ± 56.9	112.2 ± 21.6	13.723	<0.001^*^
Day 14	46.3 ± 5.9	46.0 ± 14.9	72.0 ± 21.1	56.9 ± 8.9	5.262	<0.001^*^
AST (U/L)						
Pre-	32.6 ± 4.7	32.8 ± 4.5	31.1 ± 3.1	30.0 ± 2.3	0.969	0.421
Day 1	62.6 ± 10.1	351.1 ± 94.7	499.2 ± 115.7	383.7 ± 73.4	39.569	<0.001^*^
Day 7	51.0 ± 7.9	163.8 ± 60.2	207.5 ± 35.0	175.0 ± 20.0	27.894	<0.001^*^
Day 14	51.8 ± 6.9	68.3 ± 25.7	101.4 ± 11.4	85.0 ± 16.1	13.363	<0.001^*^
TB (μmol/L)						
Pre-	20.2 ± 2.9	19.4 ± 2.2	20.8 ± 2.1	20.3 ± 1.7	0.461	0.712
Day 1	23.1 ± 1.8	30.1 ± 2.7	35.8 ± 2.9	28.5 ± 2.6	33.243	<0.001^*^
Day 7	21.9 ± 2.4	31.2 ± 4.4	39.8 ± 4.6	29.9 ± 3.0	29.868	<0.001^*^
Day 14	28.3 ± 2.9	25.4 ± 4.5	29.3 ± 2.1	26.3 ± 2.1	2.211	0.115

ALT: alanine aminotransaminase; AST: aspartate aminotransaminase; TB: total bilirubin. Data are presented as mean ± standard deviation. *p < 0.05 indicates a significant difference.

**FIGURE 3:**
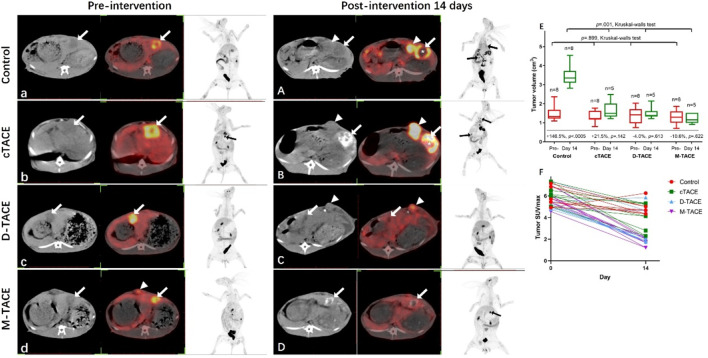
Volumetric and metabolic tumor response assessment. Representative images **(a–d and A–D)**: Non-contrast CT (left) and corresponding ^18^F-FDG PET/CT fusion images (right) obtained at baseline **(a–d)** and day 14 **(A–D)** for each group. White arrows indicate the primary intrahepatic VX2 tumor; triangles mark subcutaneously implanted tissue. In the day-14 images, asterisks (*) denote zones of spontaneous intratumoral necrosis, and triangles indicate sites of intrahepatic metastasis or residual subcutaneous growth. **(E)** Box plot of the percentage change in tumor volume from baseline to day 14 (Kruskal–Wallis test, *p* = 0.001). **(F)** Scatter plot of individual tumor SUVmax values at baseline versus day 14. Imaging: n = 5/group.

Assessment of morphologic tumor response via CT volumetry revealed effective growth control by all TACE modalities (Figure 3E). Untreated control tumors exhibited rapid progression (median volume increase of 146.5%, *p* < 0.0005). In contrast, cTACE resulted in disease stabilization, while both D-TACE and M-TACE induced actual tumor regression. The greatest degree of volumetric reduction was observed in the M-TACE group (10.6% median reduction). Consequently, inter-group differences in final tumor volume were statistically significant (*p* = 0.001).

### *In Vivo* metabolic response and metastatic suppression

^18^F-FDG PET/CT demonstrated a striking differential metabolic response to therapy (Figure 3; Table 3). Pre-procedural tumor metabolic activity, assessed by SUVmax, was comparable across all groups (*p* = 0.720). At day 14, a complete metabolic response, defined as the absence of visually discernible ^18^F-FDG uptake within the tumor bed, was achieved in all animals in the M-TACE cohort. Quantitatively, the mean post-procedural SUV ∼ max∼ in the M-TACE group (1.77 ± 0.33) was significantly lower than that in the cTACE (3.96 ± 1.23) and D-TACE (3.25 ± 1.68) groups and approached the background level of normal liver parenchyma. In contrast, residual metabolically active tissue was evident at the tumor periphery in subsets of the cTACE (3/5) and D-TACE (2/5) groups. The M-TACE strategy also demonstrated potent suppression of metastasis, with no intrahepatic or pulmonary metastatic deposits were identified (0/5), whereas metastatic rates were 20% (1/5) in both the cTACE and D-TACE groups and 75% (6/8) in the control group.

**TABLE 3:** Pre- and post-procedural metabolic activity and metastatic incidence.

​	Control	cTACE	D-TACE	M-TACE	F value	*p* value
Pre- SUVmax	6.01 ± 0.85	6.19 ± 0.75	5.56 ± 0.94	5.74 ± 0.80	0.451	0.720
Post- SUVmax	4.93 ± 0.71	3.96 ± 1.23	3.25 ± 1.68	1.77 ± 0.33	5.695	0.008*
Intrahepatic or lung metastasis (%)	6(72.5)	1(20.0)	1(20.0)	0	​	​

SUVmax: ^18^F-FDG, maximum standardized uptake value; presented as mean ± SD., Pre-: preoperative; Post-: postoperative.

### Histopathological correlates and embolic distribution

**FIGURE 4:**
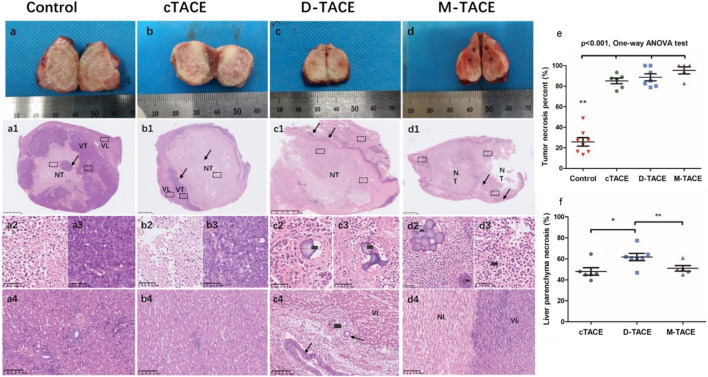
Histopathological evaluation of tumor and liver parenchyma. **(a–d)** Macroscopic appearance of resected liver specimens from each group. **(a1–d1)** Corresponding low-magnification H&E-stained whole-tumor sections. Key regions are labeled: NT, necrotic tumor; VT, viable tumor; VL, viable liver parenchyma. The scale bar is indicated. Dashed boxes outline areas shown at higher magnification below. **(a2–a4, b2–b4)** High-magnification views from the control **(a)** and cTACE **(b)** groups, detailing the interface between necrotic tumor, residual viable tumor, and adjacent liver. **(c2–c4, d2–d4)** High-magnification views from D-TACE **(c)** and M-TACE **(d)** groups. Arrows indicate drug-eluting microspheres within vessels of the necrotic tumor region. The adjacent viable liver parenchyma is also visible. **(e)** Quantitative analysis of tumor necrosis percentage. **p < 0.0001 vs. Control. **(f)** Quantitative analysis of peritumoral liver necrosis percentage. *p < 0.05, **p < 0.01 (one-way ANOVA).

Histological evaluation at the terminal endpoint corroborated the imaging findings (Figure 4). The M-TACE protocol induced the most extensive tumor necrosis (95.4% ± 6.7%), which was significantly greater than that in controls (25.8% ± 10.9%, p < 0.001) and numerically superior to both cTACE (85.1% ± 6.2%) and D-TACE (88.6% ± 8.4%). Notably, despite its potent tumoricidal effect, M-TACE resulted in a significantly lower burden of peritumoral liver parenchyma necrosis (50.9% ± 6.0%) compared to D-TACE (61.7% ± 9.0%, p = 0.039).

**FIGURE 5:**
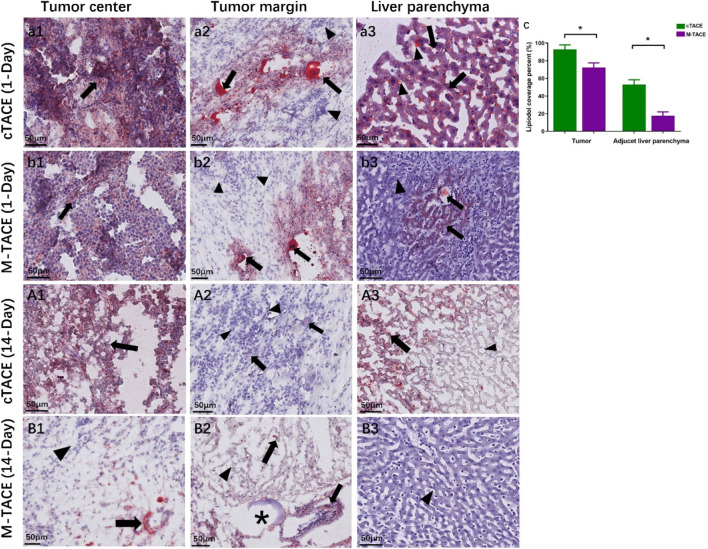
Lipiodol deposition pattern and its pathological correlation. **(a1–a3)** cTACE group: Dense, confluent Lipiodol deposition (red, arrows) is seen throughout the tumor and extending into the peritumoral liver parenchyma. Areas without Lipiodol are indicated by arrowheads. **(b1–b3)** M-TACE group: Lipiodol deposition is patchy and more confined to the tumor region, with minimal peritumoral spread. **(A1–A3)** cTACE histologic correlation: Necrosis (on H&E stain) is largely confined to areas of Lipiodol deposition (colocalization), while viable tumor cells persist in Lipiodol-sparse regions (arrowheads). **(B1–B3)** M-TACE histologic correlation: Diffuse tumor necrosis is present irrespective of Lipiodol deposition pattern. Drug-eluting microspheres (stars) are visible within tumor vasculature. **(C)** Quantification: The percentage area covered by Lipiodol was significantly lower in the M-TACE group compared to the cTACE group at 1 day post intervention, both within the tumor and in the peritumoral liver parenchyma (*p < 0.05). Scale bar: 50 μm.

Analysis of lipiodol distribution via Oil Red O staining revealed distinct patterns between groups (Figure 5). On day 1, lipiodol coverage within the tumor was significantly higher in the cTACE group (92.9% ± 7.8%) than in the M-TACE group (72.2% ± 7.4%; *p* < 0.001). This differential targeting was more pronounced in the peritumoral liver parenchyma, where lipiodol deposition measured 52.9% ± 10.7% for cTACE versus 22.1% ± 7.9% for M-TACE (*p* < 0.001).

By day 14, the spatial relationship between lipiodol and tissue necrosis differed between groups. In the cTACE group, extensive necrosis was primarily colocalized with areas of initial lipiodol deposition, while viable tumor tissue persisted at the tumor margins where lipiodol was minimal or absent. In contrast, the M-TACE group exhibited confluent tumor necrosis that extended uniformly across regions both with and without evident lipiodol deposition. Lipiodol deposition in the adjacent liver parenchyma was markedly reduced in both groups at this later time point.

### Tumor microenvironment and proliferative activity

**FIGURE 6:**
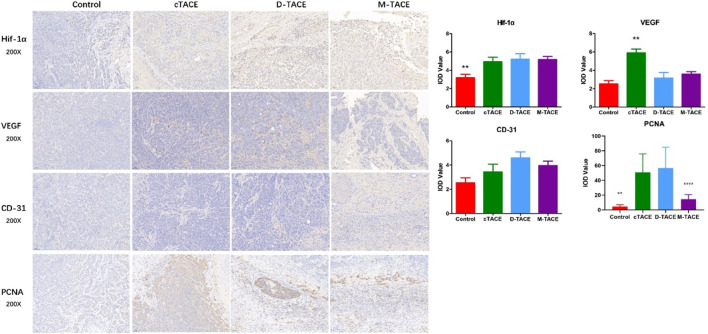
Immunohistochemical analysis of the tumor microenvironment. Representative photomicrographs (200× magnification) and corresponding semi-quantitative analysis of key biomarkers: hypoxia-inducible factor 1-alpha (HIF-1α), vascular endothelial growth factor (VEGF), cluster of differentiation 31 (CD31), and proliferating cell nuclear antigen (PCNA). The bar graph depicts the mean integrated optical density (IOD) for each marker. VEGF expression was significantly increased in the cTACE group compared to all other groups. PCNA expression was markedly elevated in both the cTACE and D-TACE groups relative to the control and M-TACE groups. **p < 0.01, ***p < 0.001 (one-way ANOVA with *post hoc* test). Scale bars: 50 μm (applied to all photomicrographs, positioned in the lower right corner).

Immunohistochemical evaluation provided mechanistic insights into the tumor biological response following different embolization protocols (Figure 6). While all TACE interventions induced comparable levels of intra-tumoral hypoxia, as evidenced by significant upregulation of hypoxia-inducible factor 1-alpha (HIF-1α) compared to controls (p = 0.01), only cTACE triggered a significant surge in vascular endothelial growth factor (VEGF) expression (p < 0.001). No significant inter-group differences were detected in CD31 expression.

Analysis of proliferative activity via proliferating cell nuclear antigen (PCNA) revealed a crucial distinction. Proliferative activity was significantly elevated in both the cTACE and D-TACE groups compared to controls (p < 0.01). In contrast, PCNA expression in the M-TACE group remained statistically indistinguishable from baseline control levels (p = 0.375) and was significantly lower than in both the cTACE and D-TACE groups (p < 0.01).

## Discussion

This preclinical study demonstrates that a sequentially staged co-delivery strategy, M-TACE, effectively addresses the historical incompatibility between lipiodol and drug-eluting microspheres in practice. By temporally segregating their administration, M-TACE allows for the independent deployment of the complementary pharmacologic strengths of each agent—lipiodol’s immediate capillary-level blockade and microspheres’ sustained drug release—culminating in a synergistic enhancement of antitumor efficacy. The protocol achieved near-total metabolic suppression and the highest degree of histologic necrosis, while concurrently demonstrating a more favorable local hepatic safety profile (as assessed by peritumoral necrosis and transaminase levels) than microsphere-alone embolization. We note that the systemic safety profile of M-TACE, particularly regarding potential extrahepatic toxicities of epirubicin such as cardiotoxicity or myelosuppression, was not evaluated in this liver-focused model and warrants investigation in future studies.

Our findings offer a mechanistic substantiation for emerging clinical observations. A recent multicenter study reported improved tumor response when lipiodol was combined with drug-eluting beads compared to beads alone (Ji et al., 2024). The observed benefits of M-TACE may be attributed to its distinct pharmacokinetic profile. While the systemic drug exposure over the initial 24 h (AUC∼0–24 h∼) was comparable across all TACE groups, this likely reflects the predominant contribution of the early, rapid-release phase to the total area within this limited sampling window. The 24-h timeframe may be insufficient to fully capture potential differences in the prolonged, terminal elimination phase governed by the sustained-release kinetics of drug-eluting microspheres. M-TACE achieved a significantly lower Cmax than cTACE, indicating a reduced risk of acute systemic toxicity. Clinical studies have shown that epirubicin-induced acute toxicities, including myelosuppression and cardiotoxicity, are dose-dependent and correlate with peak plasma concentrations. For example, plasma Cmax values exceeding 200 ng/mL have been associated with an increased incidence of grade 3/4 neutropenia (Robert, 1994), and cumulative doses >900 mg/m^2^ are linked to cardiotoxicity (Ryberg et al., 2008). The Cmax observed in our cTACE group (276 ng/mL) approaches this threshold, whereas the lower Cmax in M-TACE (92 ng/mL) suggests a reduced risk of such acute adverse effects. Extending the pharmacokinetic monitoring to 3 or 7 days might better differentiate the profiles, cumulative systemic exposure with M-TACE, further highlighting its pharmacokinetic advantage.

Crucially, the direct measurement of intratumoral epirubicin concentration demonstrated that M-TACE maintained drug levels equivalent to D-TACE at both early (Day 1) and late (Day 14) time points. This key finding provides direct evidence that prior lipiodol injection did not impair the loading or elution kinetics of the subsequent drug-eluting microspheres. Future experimental designs incorporating concurrent late-term plasma sampling will be valuable to further characterize the complete pharmacokinetic profile. The achieved profile of “low systemic peak, high and sustained local” drug exposure underlies the profound tumor necrosis (95.4%) induced by M-TACE.

The significantly lower peritumoral liver necrosis observed in M-TACE (50.9% ± 6.0%) compared to D-TACE (61.7% ± 9.0%, p = 0.039) suggests improved targeting specificity and reduced off-target embolization with the sequential strategy. This difference can be attributed to the initial lipiodol emulsion in M-TACE, administered at a lower volume (0.2 mL vs. 1.0 mL in cTACE), selectively deposits in the hypervascular tumor bed due to the “lipiodol trapping” phenomenon.

This potent cytotoxic and embolic effect was validated through multimodal assessment. It led to significant tumor volume reduction morphologically, complete metabolic remission on ^18^F-FDG PET/CT functionally, and near-total necrosis histopathologically. The superior efficacy of M-TACE is likely rooted in its stepwise mechanism, which addresses the anatomical and pharmacokinetic limitations inherent to both cTACE and D-TACE. The initial bolus of lipiodol emulsion penetrates the tumor microcirculation and portal venules, achieving an initial cytotoxic effect and demarcating a treatment margin. Lipiodol not only aids in visualizing and tracking the tumor but also deposits within tumor tissues, facilitating the delivery of cytotoxic drugs into tumor cells (Imaeda et al., 1993; Shin, 2009). Current evidence suggests that targeting the multiple blood supplies to liver tumors—including the hepatic artery, portal vein, and arterial collaterals—is associated with better local tumor control after TACE (Goseki et al., 1995; Kuroda et al., 1991; Miyayama et al., 2006).

The subsequent infusion of drug-eluting microspheres may serve a dual purpose: it potentially secures this lipiodol depot by reducing arterial inflow and “washout,” while possibly establishing a second, prolonged phase of localized chemotherapy (Idee and Guiu, 2013; Kan and Madoff, 2008). This concept is supported by the sustained intratumoral drug concentrations observed at day 14. The equivalent intratumoral epirubicin concentration in the M-TACE and D-TACE groups, unequivocally prove that prior lipiodol administration does not impair the microsphere kinetics, enabling sustained local drug exposure—a key factor behind the observed necrosis and metabolic arrest.

Beyond direct cytotoxic effects, M-TACE favorably modulated the post-embolization tumor microenvironment. While all TACE procedures induced comparable hypoxia (elevated HIF-1α), only cTACE triggered a significant surge in VEGF expression, suggesting a potent pro-angiogenic rebound that may fuel local recurrence (Chung et al., 2025; Llovet et al., 2021; Pinto et al., 2024). Crucially, PCNA was markedly elevated after cTACE and D-TACE but not after M-TACE. This triad of findings—attenuated angiogenic stimulus coupled with suppressed proliferation—suggests that M-TACE disrupts the adaptive survival circuitry often activated by incomplete embolization, thereby explaining its superior long-term necrotic and anti-metastatic outcomes. Thus, consistent with prior work (Li et al., 2024), this biological profile underpins the enhanced necrosis and absence of metastasis observed with M-TACE.

Prior attempts to combine these agents involved physical formulation changes, such as freeze-drying beads (DC Beads) to improve lipiodol compatibility (Caine et al., 2019). Lyophilized format allows for initial mixing with lipiodol oil which can penetrate the DEBs structure and aid in the emulsion compatibility. In contrast, our sequential approach provides a practical solution without altering the physicochemical properties of the microspheres. Our findings extend prior investigations by demonstrating that therapeutic synergy can be achieved by optimizing the temporal delivery of each component, challenging the conventional either-or paradigm in TACE technique selection.

While the modified Response Evaluation Criteria in Solid Tumors (mRECIST) is routinely recommended for clinical assessing tumor response following locoregional therapy (European Association for the Study of the Liver. Electronic address & European Association for the Study of the L, 2018), this study utilized ^18^F-FDG PET/CT for comprehensive evaluation. This multimodal imaging approach is particularly valuable in the context of embolization, as it integrates anatomical and functional data. The CT component accurately delineates lipiodol deposition, while the concomitant PET scan identifies metabolically active viable tissue within or adjacent to these embolized areas—a distinction often obscured on contrast-enhanced CT alone (Okuma et al., 2006; Xiang et al., 2020). In this study, PET/CT was instrumental in detecting residual metabolically active tumor at the margins following cTACE and D-TACE, and in confirming the absence of such activity after M-TACE. Furthermore, it provided definitive evidence regarding the presence or absence of extrahepatic metastatic disease, thereby offering a more complete portrait of treatment efficacy.

Several limitations warrant consideration. The sample size, while adequate for this proof-of-concept study, may limit the power to detect subtler differences between the TACE groups. The VX2 tumor model, though standard, does not fully recapitulate the complex vasculature and cirrhosis of human HCC. This difference may significantly influence embolic distribution, lipiodol retention dynamics, and the development of compensatory collateral circulation post-embolization. Validation in more complex models, such as those incorporating liver fibrosis or patient-derived xenografts, and ultimately in clinical trials, is essential to confirm its efficacy and safety in the context of human HCC with cirrhosis. Furthermore, the optimal dosing ratio and timing interval between lipiodol and microspheres require further parametric investigation to establish a standardized protocol. Extended PK sampling to 72–96 h would be valuable for future studies to fully characterize the terminal elimination phase. We have framed this as an important direction for subsequent investigation.

In conclusion, this study introduces a rational and effective technical evolution in TACE. The sequential M-TACE strategy successfully decouples the delivery of two powerful yet incompatible therapeutic agents, permitting their sequential application and synergy while preserving the pharmacokinetic profile of either. By delivering comprehensive tumor necrosis, complete metabolic remission, and reduced metastatic incidence alongside a refined local hepatic safety profile, M-TACE presents a compelling translational framework. Future clinical studies are warranted to validate these promising preclinical results and to refine this approach for the treatment of intermediate-stage HCC.

## Data Availability

The raw data supporting the conclusions of this article will be made available by the authors, without undue reservation.

## References

[B1] Caine M. Chung T. Kilpatrick H. Bascal Z. Willis S. Tang Y. (2019). Evaluation of novel formulations for transarterial chemoembolization: combining elements of lipiodol emulsions with drug-eluting beads. Theranostics 9 (19), 5626–5641. 10.7150/thno.34778 31534507 PMC6735388

[B2] Casadaban L. C. Minocha J. Bui J. T. Knuttinen M. G. Ray C. E. Jr. Gaba R. C. (2016). Conventional ethiodized oil transarterial chemoembolization for treatment of hepatocellular carcinoma: contemporary single-center review of clinical outcomes. AJR Am. J. Roentgenol. 206 (3), 645–654. 10.2214/AJR.15.14758 26901023

[B3] Chuang Y. H. Cheng Y. F. Tsang L. L. Ou H. Y. Hsu H. W. Lim W. X. (2023). Efficacy and safety of combined ethanol-lipiodol mixture and drug-eluting bead TACE for large HCC. J. Hepatocell. Carcinoma 10, 81–90. 10.2147/JHC.S398434 36685112 PMC9850831

[B4] Chung S. W. Kim J. S. Choi W. M. Choi J. Lee D. Shim J. H. (2025). Synergistic effects of transarterial chemoembolization and lenvatinib on HIF-1alpha ubiquitination and prognosis improvement in hepatocellular carcinoma. Clin. Cancer Res. 31 (10), 2046–2055. 10.1158/1078-0432.CCR-24-1228 39992640

[B5] Clouet J. Audureau D. Lefranc B. Maillard N. Guile R. Frampas E. (2014). Medico-economic study of the management of hepatocellular carcinoma by chemo-embolization. Diagn Interv. Imaging 95 (4), 427–434. 10.1016/j.diii.2013.10.012 24231346

[B6] European Association for the Study of the Liver. Electronic address & European Association for the Study of the L (2018). EASL clinical practice guidelines: Management of hepatocellular carcinoma. J. Hepatol. 69 (1), 182–236. 10.1016/j.jhep.2018.03.019 29628281

[B7] Fuchs K. Duran R. Denys A. Bize P. E. Borchard G. Jordan O. (2017). Drug-eluting embolic microspheres for local drug delivery - state of the art. J. Control Release 262, 127–138. 10.1016/j.jconrel.2017.07.016 28710006

[B8] Goseki N. Nosaka T. Endo M. Koike M. (1995). Nourishment of hepatocellular carcinoma cells through the portal blood flow with and without transcatheter arterial embolization. Cancer 76 (5), 736–742. 10.1002/1097-0142(19950901)76:5<736::aid-cncr2820760505>3.0.co;2-q 8625174

[B9] Guo J. Huang J. Huang Z. Hu D. Tan H. Wang Y. (2025). Tumor vessel-adaptable adhesive and absorbable microspheres for sustainable transarterial chemoembolization therapy. Nat. Commun. 16 (1), 6239. 10.1038/s41467-025-61621-4 40624007 PMC12234964

[B10] Hong K. Khwaja A. Liapi E. Torbenson M. S. Georgiades C. S. Geschwind J. F. (2006). New intra-arterial drug delivery system for the treatment of liver cancer: preclinical assessment in a rabbit model of liver cancer. Clin. Cancer Res. 12 (8), 2563–2567. 10.1158/1078-0432.CCR-05-2225 16638866

[B11] Idee J. M. Guiu B. (2013). Use of lipiodol as a drug-delivery system for transcatheter arterial chemoembolization of hepatocellular carcinoma: a review. Crit. Rev. Oncol. Hematol. 88 (3), 530–549. 10.1016/j.critrevonc.2013.07.003 23921081

[B12] Ikeda M. Arai Y. Inaba Y. Tanaka T. Sugawara S. Kodama Y. (2022). Conventional or drug-eluting beads? Randomized controlled study of chemoembolization for hepatocellular carcinoma: JIVROSG-1302. Liver Cancer 11 (5), 440–450. 10.1159/000525500 36158586 PMC9485929

[B13] Imaeda T. Yamawaki Y. Seki M. Goto H. Iinuma G. Kanematsu M. (1993). Lipiodol retention and massive necrosis after lipiodol-chemoembolization of hepatocellular carcinoma: correlation between computed tomography and histopathology. Cardiovasc Interv. Radiol. 16 (4), 209–213. 10.1007/BF02602962 8402781

[B14] Ji K. Shi Y. Liang Z. Zhang C. Jing L. Xu T. (2024). Lipiodol combined with drug-eluting beads Versus drug-eluting beads alone for transarterial chemoembolization of hepatocellular carcinoma: a multicenter study. Acad. Radiol. 31 (12), 4912–4922. 10.1016/j.acra.2024.05.033 38866689

[B15] Kan Z. Madoff D. C. (2008). Liver anatomy: microcirculation of the liver. Semin. Interv. Radiol. 25 (2), 77–85. 10.1055/s-2008-1076685 21326548 PMC3036477

[B16] Kuroda C. Sakurai M. Monden M. Marukawa T. Hosoki T. Tokunaga K. (1991). Limitation of transcatheter arterial chemoembolization using iodized oil for small hepatocellular carcinoma. A study in resected cases. Cancer 67 (1), 81–86. 10.1002/1097-0142(19910101)67:1<81::aid-cncr2820670116>3.0.co;2-h 1845939

[B17] Li Y. Ge X. Li Z. Zhou Z. Wu K. Li Y. (2024). Application of temperature-sensitive liquid embolic agent loaded with oxaliplatin in the TACE procedure for rabbit VX2 gastric cancer. Drug Deliv. Transl. Res. 14 (3), 705–717. 10.1007/s13346-023-01425-5 37668861

[B18] Llovet J. M. De Baere T. Kulik L. Haber P. K. Greten T. F. Meyer T. (2021). Locoregional therapies in the era of molecular and immune treatments for hepatocellular carcinoma. Nat. Rev. Gastroenterol. Hepatol. 18 (5), 293–313. 10.1038/s41575-020-00395-0 33510460

[B19] Miyayama S. Matsui O. Taki K. Minami T. Ryu Y. Ito C. (2006). Extrahepatic blood supply to hepatocellular carcinoma: angiographic demonstration and transcatheter arterial chemoembolization. Cardiovasc Interv. Radiol. 29 (1), 39–48. 10.1007/s00270-004-0287-y 16328697

[B20] Okuma T. Matsuoka T. Okamura T. Wada Y. Yamamoto A. Oyama Y. (2006). 18F-FDG small-animal PET for monitoring the therapeutic effect of CT-guided radiofrequency ablation on implanted VX2 lung tumors in rabbits. J. Nucl. Med. 47 (8), 1351–1358. 16883016

[B21] Pinto E. Pelizzaro F. Cardin R. Battistel M. Palano G. Bertellini F. (2024). HIF-1alpha and VEGF as prognostic biomarkers in hepatocellular carcinoma patients treated with transarterial chemoembolization. Dig. Liver Dis. 56 (5), 872–879. 10.1016/j.dld.2023.09.019 37783655

[B22] Reig M. Sanduzzi-Zamparelli M. Forner A. Rimola J. Ferrer-Fabrega J. Burrel M. (2025). BCLC strategy for prognosis prediction and treatment recommendations: the 2025 update. J. Hepatol. 84, 631–654. 10.1016/j.jhep.2025.10.020 41151697

[B23] Robert J. (1994). Clinical pharmacokinetics of epirubicin. Clin. Pharmacokinet. 26 (6), 428–438. 10.2165/00003088-199426060-00002 8070217

[B24] Ryberg M. Nielsen D. Cortese G. Nielsen G. Skovsgaard T. Andersen P. K. (2008). New insight into epirubicin cardiac toxicity: competing risks analysis of 1097 breast cancer patients. J. Natl. Cancer Inst. 100 (15), 1058–1067. 10.1093/jnci/djn206 18664656

[B25] Shin S. W. (2009). The current practice of transarterial chemoembolization for the treatment of hepatocellular carcinoma. Korean J. Radiol. 10 (5), 425–434. 10.3348/kjr.2009.10.5.425 19721826 PMC2731859

[B26] Xiang Z. Q. Imani S. Hu Y. Ding R. L. Pang H. W. Chen Y. (2020). Comparison of different images in gross target volume delineating on VX2 nasopharyngeal transplantation tumor models. J. Cancer 11 (5), 1104–1114. 10.7150/jca.36076 31956357 PMC6959086

[B27] Yamamoto A. Imai S. Kobatake M. Yamashita T. Tamada T. Umetani K. (2006). Evaluation of tris-acryl gelatin microsphere embolization with monochromatic X rays: comparison with polyvinyl alcohol particles. J. Vasc. Interv. Radiol. 17 (11), 1797–1802. 10.1097/01.RVI.0000243614.87529.b0 17142710

[B28] Zhou T. Y. Tao G. F. Zhou G. H. Zhang Y. L. Zhu T. Y. Chen S. Q. (2024). Comparison of drug-eluting bead with conventional transcatheter arterial chemoembolization for hepatocellular carcinoma with portal vein tumor thrombus: a randomized clinical trial. Int. J. Surg. 110 (9), 5527–5537. 10.1097/JS9.0000000000001691 38775550 PMC11392094

