# Correction: Sequential and synergistic delivery of lipiodol and drug-eluting microspheres circumvents incompatibility to enhance targeted chemoembolization

**DOI:** 10.3389/fphar.2026.1845892

**Published:** 2026-04-15

**Authors:** Frontiers Production Office

**Affiliations:** Frontiers Media SA, Lausanne, Switzerland

**Keywords:** chemoembolization, epirubicin, hepatocellular carcinoma, lipiodol, liver function, microsphere, rabbit, tumor necrosis

Affiliation of Reviewer Kaushik Neogi was erroneously given as “Deemed to be University, India”. The correct affiliation is “SVKM’s NMIMS (Deemed to be University), India.”

The original article has been updated.

