# Supplementary material for: Sequential and synergistic delivery of lipiodol and drug-eluting microspheres circumvents incompatibility to enhance targeted chemoembolization
Source: Front Pharmacol. 2026 Mar 30;17:1800481. doi: 10.3389/fphar.2026.1800481 (PMC13071007; doi:10.3389/fphar.2026.1800481)
Supplement: Supplementary file 1 [file Supplementaryfile1.docx]

**Appendix information**

**1. Pharmacokinetic Analysis by HPLC-MS/MS**

Serum and tumor tissue concentrations of epirubicin were quantified using a validated high-performance liquid chromatography-tandem mass spectrometry (HPLC-MS/MS) method. The analysis was performed on a system consisting of a TSQ Quantum Ultra mass spectrometer (Thermo Fisher Scientific) coupled with a Dionex UltiMate 3000 RSLC Nano system (Thermo Fisher Scientific).

- Standard Curve and Sample Preparation: A seven-point calibration curve was prepared by spiking blank rabbit serum with a working epirubicin standard solution (Shanghai Kewel Chemical Technology Co.), yielding concentrations from 5 to 5000 ng/mL. The lower limit of quantification (LLOQ) was established at 5 ng/mL. Plasma samples were deproteinized using methanol prior to analysis. Tumor tissues were weighed, homogenized in ice-cold phosphate-buffered saline (PBS; 1:5, w/v), and centrifuged. The supernatant was collected, diluted (1:10, v/v) with PBS, and processed identically to plasma samples.
- Chromatographic Conditions: Separation was achieved using a Thermo Hypersil GOLD C18 column (2.1 × 100 mm, 1.9 μm). The mobile phase flow rate was set at 0.5 mL/min, and the injection volume was 5 μL for both standards and samples.
- Pharmacokinetic Calculations: The maximum observed plasma concentration (Cmax) and the time to Cmax (Tmax) were recorded directly. The area under the plasma concentration-time curve from 0 to 24 hours (AUC~0–24h~) was calculated using the linear trapezoidal rule: AUC_0–24h_ = Σ [ (Ci~ + Ci+1) / 2 × (ti+1– ti)], where Ci is the plasma concentration at time ti.

**2. PET/CT Image Acquisition and Analysis**

¹⁸F-FDG PET/CT imaging was performed at baseline and 14 days post-intervention. Detailed imaging parameters are provided in the main Methods section. For analysis, PET images were co-registered with the corresponding CT images for anatomical reference. A volume of interest (VOI) was manually delineated around the entire tumor on the CT images, guided by baseline contrast-enhanced angiography. This VOI was applied to the co-registered PET data to extract functional parameters. The maximum standardized uptake value (SUVmax) and mean SUV (SUVmean) within the tumor VOI were calculated. A significant reduction in SUV was considered indicative of treatment-induced metabolic response.

**3. Oil Red O Staining and Quantitative Analysis**

To evaluate lipiodol distribution, tumor samples from the cTACE and M-TACE groups were collected at day 1 and day 14, snap-frozen in dry ice, and stored at –80°C. Fresh-frozen tissue sections were prepared using a cryostat and stained with Oil Red O (ORO; Sigma-Aldrich, St. Louis, MO, USA) according to a standard protocol.

For quantitative analysis, entire ORO-stained slides were digitized using a high-resolution slide scanner. The digitized images were evaluated under high-power magnification (×200). Lipiodol deposition within the tumor and the adjacent liver parenchyma was quantified by calculating the percentage of ORO-positive stained area relative to the total area of the respective tissue field, using digital image analysis software.

Quantitative analysis of lipiodol coverage was performed using Image-Pro Plus 6.0 software (Media Cybernetics, Bethesda, MD, USA) by two independent pathologists who were blinded to group allocation. For each section, regions of interest (ROIs) were manually delineated to distinguish tumor area from peritumoral liver parenchyma. Within each ROI, lipiodol-positive area was identified using consistent color thresholding parameters (hue: 0-30 for red staining; saturation: 50-255; intensity: 50-255) applied uniformly across all sections. The percentage of lipiodol coverage was calculated as: (lipiodol-positive area / total ROI area) × 100%.

**4. Terminal half-life and local/systemic exposure ratio**

The estimated terminal half-life values (cTACE: 4.2 ± 1.1 h; D-TACE: 8.7 ± 2.3 h; M-TACE: 8.9 ± 2.1 h) were calculated using the last three time points (60, 180, and 1440 minutes) and should be interpreted as apparent terminal half-lives reflecting the combined effects of distribution and early elimination within the limited sampling window. Future studies with extended PK sampling to 72-96 hours are warranted to accurately determine the true terminal half-life of microsphere-based formulations.

Tumor-to-plasma (T/P) concentration ratios were calculated for Day 1 by dividing the intratumoral epirubicin concentration (ng/g) by the corresponding plasma concentration (ng/mL) from the same animal at the terminal 24-hour time point. Day 14 T/P ratios could not be calculated as plasma samples were not collected at this time point, in accordance with the study design prioritizing histopathological analysis.

The Day 1 tumor-to-plasma concentration ratio was significantly higher in both D-TACE (294.6 ± 42.8) and M-TACE (278.9 ± 38.4) groups compared to cTACE (58.3 ± 15.2) (p<0.001), confirming superior initial local drug sequestration with microsphere-based therapies. Importantly, the Day 1 T/P ratios were comparable between M-TACE and D-TACE (p=0.92), providing direct evidence that prior lipiodol administration did not impair early microsphere function.

**Chemicals and antibodies used in this study.**

Epirubicin hydrochloride: Pfizer Inc., New York, NY, USA

Lipiodol Ultra-Fluide: Guerbet, Villepinte, France

HepaSphere™ microspheres (20-40 μm dry state): Merit Medical, South Jordan, UT, USA

Gelatin sponge particles (150-350 μm): Alicon, Hangzhou, China

Anti-HIF-1α antibody: Abcam, Cambridge, UK

Anti-VEGF antibody: Abcam, Cambridge, UK

Anti-CD31 antibody: Abcam, Cambridge, UK

Anti-PCNA antibody: Abcam, Cambridge, UK

Oil Red O stain: Sigma-Aldrich, St. Louis, MO, USA

Hematoxylin and eosin: Sigma-Aldrich, St. Louis, MO, USA
