# Supplementary material for: Sequential and synergistic delivery of lipiodol and drug-eluting microspheres circumvents incompatibility to enhance targeted chemoembolization
Source: Front Pharmacol. 2026 Mar 30;17:1800481. doi: 10.3389/fphar.2026.1800481 (PMC13071007; doi:10.3389/fphar.2026.1800481)
Supplement: Supplementary file 2 [file Supplementaryfile2.docx]

**Supplementary Figures**

**
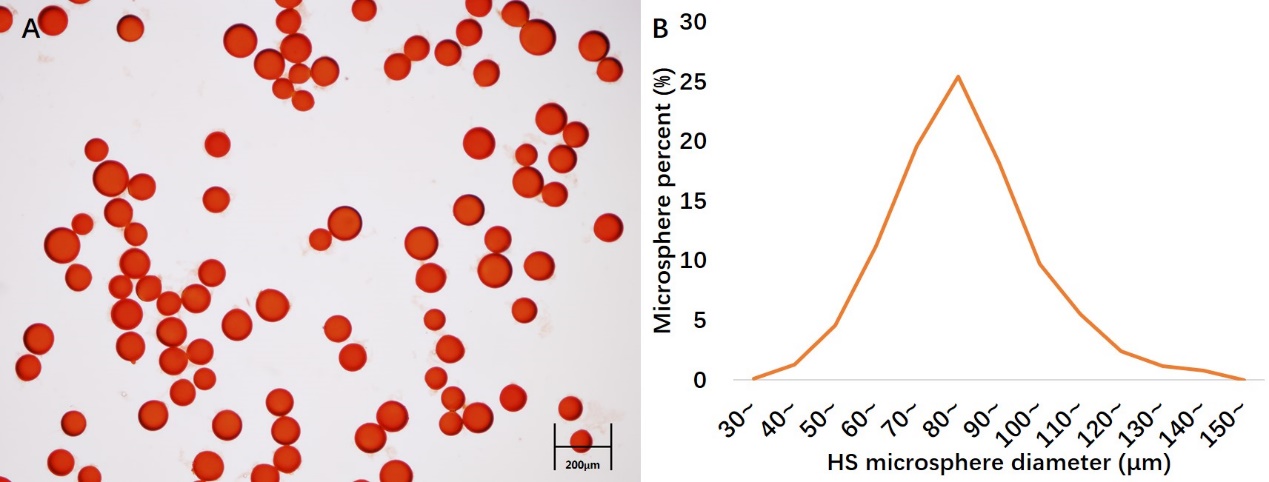
Supplement figure 1. Characterization of drug-eluting microspheres.** (A) Light micrograph of HepaSphere™ microspheres after loading with epirubicin, showing uniform spherical morphology and swelling (scale bar: 200 μm). (B) Size distribution histogram of the hydrated microspheres, showing a mean diameter of 84.2 ± 17.6 μm.


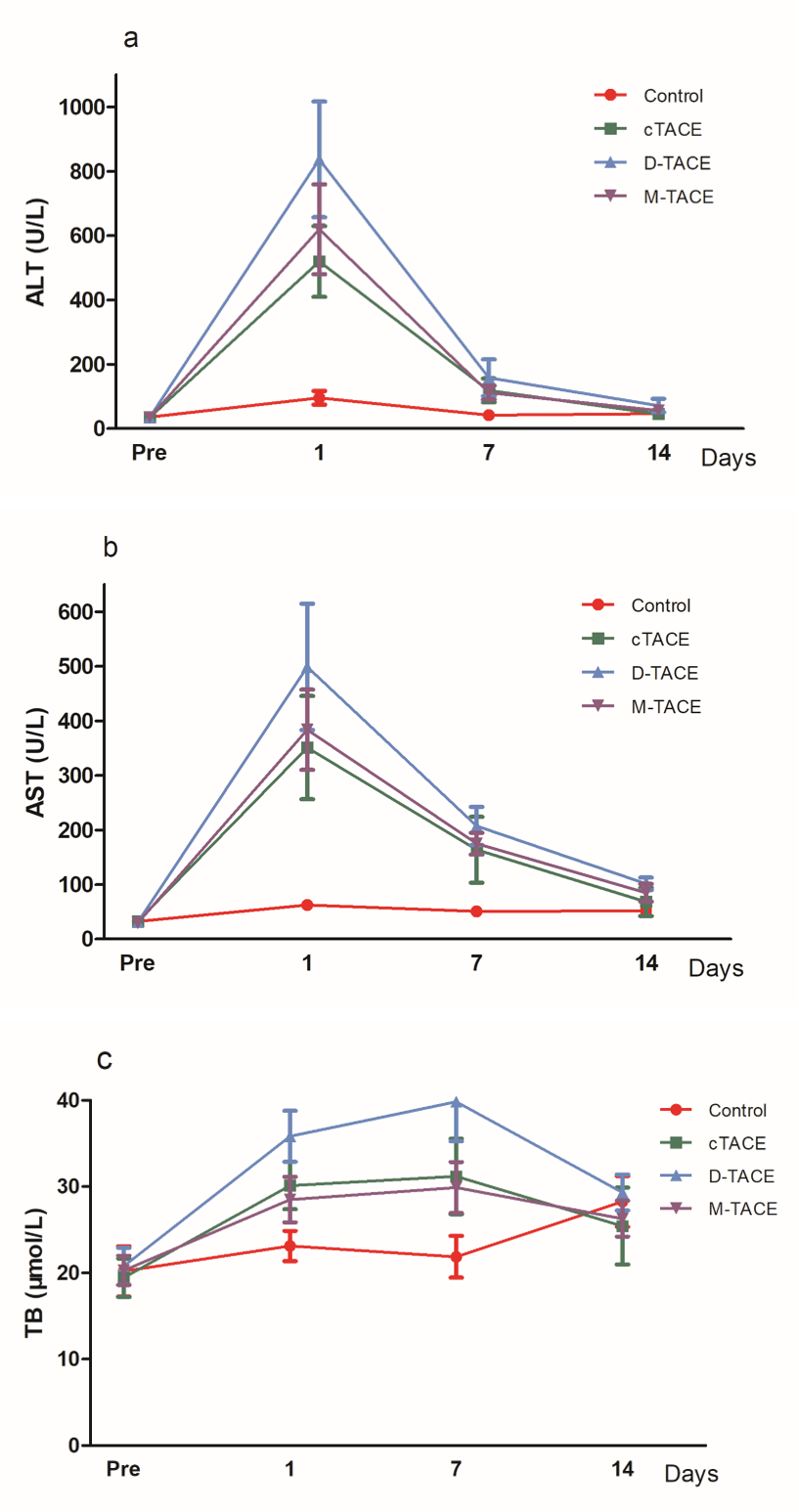


**Supplementary figure 2. Longitudinal changes in liver fuctions.** Temporal profiles of plasma (A) alanine aminotransferase (ALT), (B) aspartate aminotransferase (AST), and (C) total bilirubin (TB) levels over the 14-day study period. Data are presented as mean ± SD. The D-TACE group exhibited the most pronounced acute elevation in ALT and AST at day 1.

**Supplement figure 3**


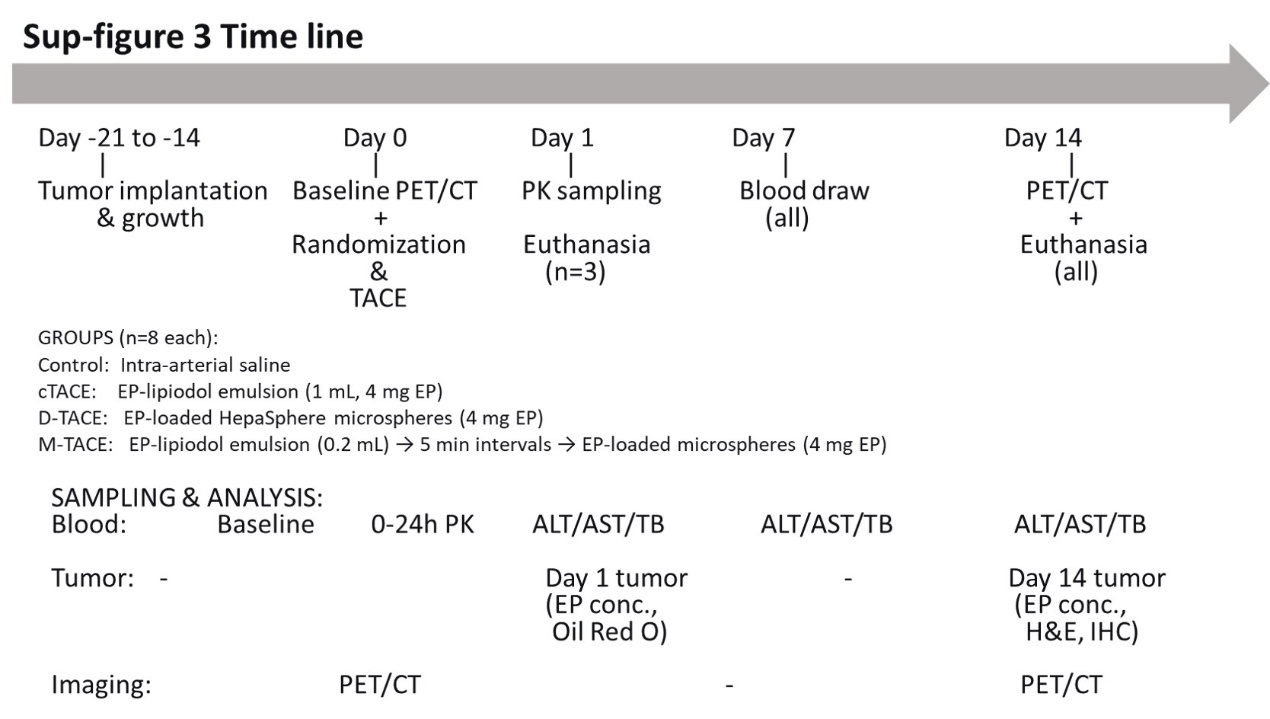


**Supplement figure 3.** Supplement figure 3. Experimental design schematic for the VX2 rabbit model.
